# Tight association of genome rearrangements with gene expression in conifer plastomes

**DOI:** 10.1186/s12870-020-02809-2

**Published:** 2021-01-08

**Authors:** Chung-Shien Wu, Edi Sudianto, Shu-Miaw Chaw

**Affiliations:** Biodiversity Research Center, Academia Sinica, Taipei, 11529 Taiwan

**Keywords:** Conifer, Plastid transcriptome, Plastomic rearrangement, Strand-specific RNAseq, RNA-editing

## Abstract

**Background:**

Our understanding of plastid transcriptomes is limited to a few model plants whose plastid genomes (plastomes) have a highly conserved gene order. Consequently, little is known about how gene expression changes in response to genomic rearrangements in plastids. This is particularly important in the highly rearranged conifer plastomes.

**Results:**

We sequenced and reported the plastomes and plastid transcriptomes of six conifer species, representing all six extant families. Strand-specific RNAseq data show a nearly full transcription of both plastomic strands and detect C-to-U RNA-editing sites at both sense and antisense transcripts. We demonstrate that the expression of plastid coding genes is strongly functionally dependent among conifer species. However, the strength of this association declines as the number of plastomic rearrangements increases. This finding indicates that plastomic rearrangement influences gene expression.

**Conclusions:**

Our data provide the first line of evidence that plastomic rearrangements not only complicate the plastomic architecture but also drive the dynamics of plastid transcriptomes in conifers.

**Supplementary Information:**

The online version contains supplementary material available at 10.1186/s12870-020-02809-2.

## Background

Conifers are a group of cone-bearing seed plants. They comprise ca. 630 species in two clades, Pinaceae (conifers I clade) and cupressophytes (conifers II clade, consisting of five families). Conifers dominate temperate forests, especially in the Northern hemisphere, and significantly contribute to photosynthesis and biomass production. They provide shelters for wildlife and important resources for humans, such as solid wood fuel, valuable timber, edible seeds, and essential oils [1].

Plastid gene transcription is a complex process, involving both prokaryotic- and eukaryotic-type systems [2]. Most plastid genes are presumably transcribed as polycistronic mRNAs which then undergo various post-transcriptional modifications [3]. These processes generate tremendously elaborate transcriptomes with an unprecedented diversity of non-coding RNAs [4], multiple loci for transcriptional initiation and termination [5, 6], a full or nearly full transcription of the genome [7, 8], and varying frequencies of RNA-editing sites [9].

Plastid genomes (plastomes) of land plants are highly conserved in their gene content and order. Functionally related genes are commonly found in clusters and are likely co-transcribed as operons [10]. These operons may be conserved due to selective constraints rather than slow rates of neutral chromosomal rearrangements [11]. However, mounting evidence indicates that many taxa, including conifers (the largest group of gymnosperms), have highly rearranged plastomes [12–14]. Some of these rearrangements resulted in disruption of canonical operons and creation of novel co-transcriptional units. An example is the disruption of *rps2* operons in *Sciadopitys* and *Callitris* [15, 16]. We have long been puzzled by these findings because it is then unclear whether plastomic rearrangements affect plastid gene transcription. If they do, what are the underlying mechanisms and consequences of such changes?

In this study, we sequenced both plastomic DNA and RNA from one representative genus in each of the six extant conifer families. Strand-specific RNA libraries have the advantage of allowing for the discrimination of sense and antisense transcripts [17]. We took advantage of this to (1) investigate the full transcription capability of both plastomic strands, (2) estimate the relative number of plastid coding and antisense transcripts, and (3) identify plastid C-to-U RNA-editing sites separately at sense and antisense transcripts in conifers. We also compared plastid gene expression levels among conifers and demonstrated a strong association between gene expression and plastomic rearrangements. We discuss possible mechanisms underlying this association.

## Results

### Both plastomic strands are fully transcribed in conifers

**Fig. 1:**
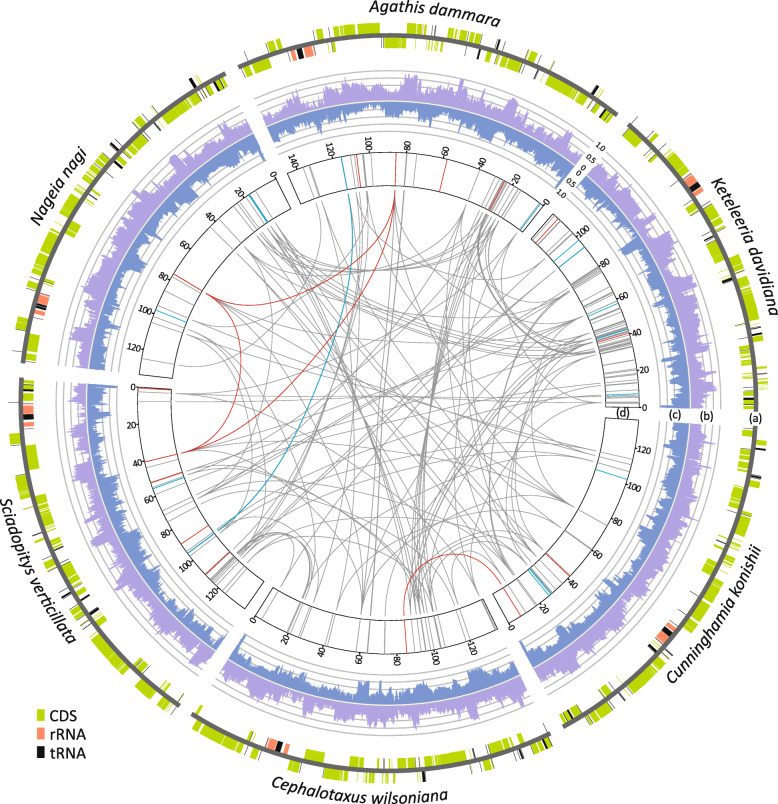
Plastid transcriptomic profiles of the six sampled conifer species. (**a**) Plastomic maps with genes in outer and inner strands transcribed clockwise and counterclockwise, respectively. Transcriptomic profiles where outer (**b**) and inner (**c**) histograms represent RNAseq coverage (read counts per base) after transformation by the formula: Log_10_ (coverage + 1) / Log_10_ (maximum coverage + 1). (**d**) Distribution of RNA-editing sites where red, blue, and grey lines denote anti-sense, silent, and non-silent editing, respectively. Shared edited sites are linked by lines

The six newly assembled plastomes are illustrated as linear molecules to facilitate pairwise comparisons (Fig. 1a). A plastomic inversion was detected in the sampled *K. davidiana* individual when it was compared to the conspecific reference (NC_011930; Fig. S1a). This polymorphic inversion is flanked by Pinaceae Type I repeats [18], which are capable of triggering homologous recombination to generate predominant and substoichiometric plastomic isomers in *K. davidiana* (Fig. S1b).

RNAseq coverage across the six sampled conifer plastomes is represented as histograms in Fig. 1. RNAseq coverage of rRNAs is low, indicating effective depletion of rRNA transcripts prior to sequencing. We also found that over 94.2% of the plastome sequences were covered by RNAs generated from a specific single DNA strand. The coverage ratio increased to over 99.9% after RNAseq reads from both strands were combined (Fig. S2). Overall, our data reveal almost full transcription of both plastomic strands, indicating that intergenic, intronic, and antisense transcripts are ubiquitous in conifer plastids.

Our data also show that CDS sense transcripts are generally more abundant than their antisense counterparts, although there are several exceptions (Fig. S3). For example, *psbN,* a photosynthetic system II gene, is located on the strand opposite to the *psbB* operon, a well-known polycistronic transcription unit that comprises four genes: *psbB*, *psbH*, *petB*, and *petD* [19]. Therefore, the transcripts antisense to *psbN* are likely overrepresented due to the strongly expressed *psbB* operon.

### Influence of plastomic rearrangements on CDS expression

**Fig. 2:**
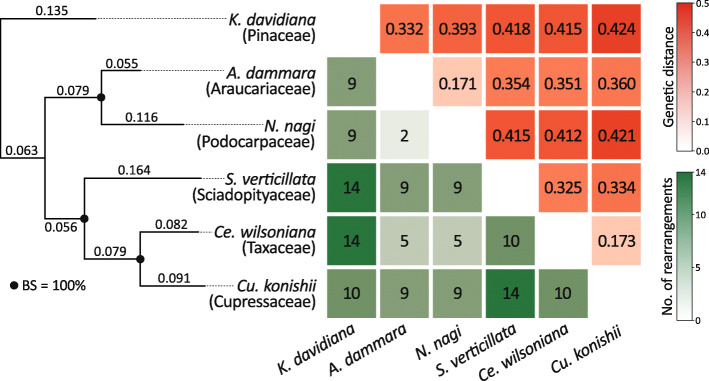
Plastomic rearrangements taken place during the conifer evolution. A maximum likelihood tree inferred from the 83 orthologous CDSs is depicted in the left panel. Families of sampled conifers are indicated in parentheses. Branch lengths used in calculating genetic distances are labelled along the tree branches. Pairwise rearrangement counts (within green squares) and genetic distances (within red squares) are shown in the right panel. BS, bootstrap support

Among the six conifer plastomes, 31 syntenic blocks were identified to estimate plastomic rearrangements (Fig. S4a). Pairwise dot-plot analyses of these six plastomes are also shown in Fig. S4b. Our comparisons reveal 2–14 rearrangements among the sampled conifers (Fig. 2). To examine whether the phylogenetic distances are associated with the frequency of plastomic rearrangements, we estimated interspecific genetic distances based on the branches of the tree inferred from the concatenation of 83 orthologous CDSs (Fig. 2). We did not find significant correlation between genetic distances and plastomic rearrangement counts (Pearson’s *ρ* = 0.375, *P* = 0.167; Fig. S5).

**Fig. 3:**
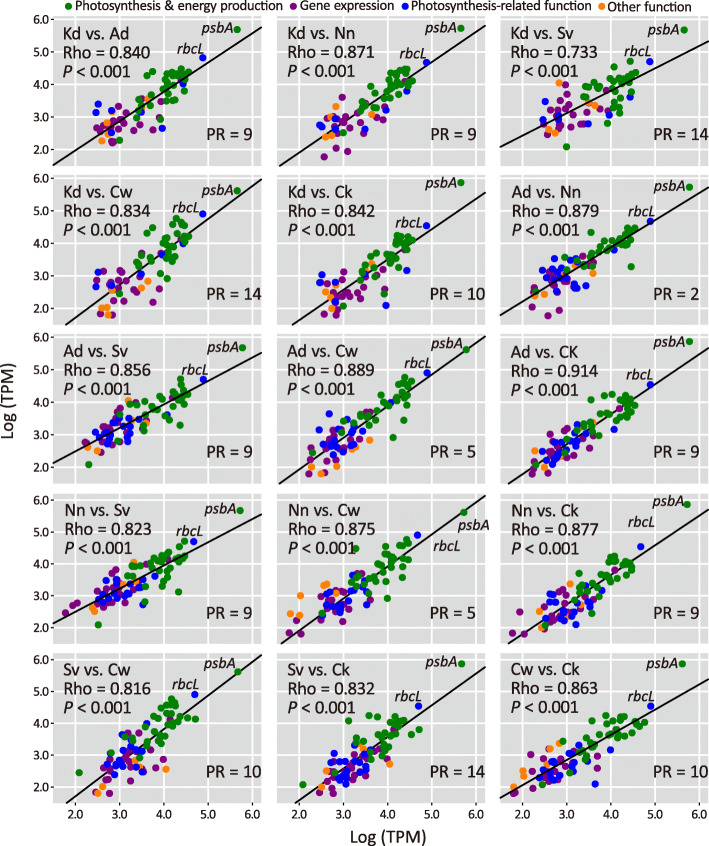
Strong correlations among plastid CDS expression levels in the sampled conifer species. Kd, *K. davidiana*; Ad, *A. dammara*; Nn, *N. nagi*; Sv, *S. verticillata*; Cw, *Ce. wilsoniana*; Ck, *Cu. konishii*; Rho (*ρ*), Pearson correlation coefficient; PR, numbers of plastomic rearrangements between species

To normalize expression levels, RNAseq reads mapped to CDSs were collected and combined to calculate transcripts per million (TPM). Figure 3 compares the TPM scores between orthologous plastid CDSs that retain equivalent functions across conifer species. We found that (1) *psbA* and *rbcL* are the two most highly expressed genes in the presence of light and (2) TPM scores of these orthologous genes are significantly correlated (Pearson’s *ρ* = 0.733 to 0.914, all *P* < 0.001), suggesting that their expression levels are strongly functionally dependent. However, these correlation coefficients are inversely associated with the number of plastomic rearrangements (PR) between species (Pearson’s *ρ* = − 0.626, *P* = 0.013; Fig. S6). Taken together, our results demonstrate that plastomic rearrangements reduce the strength of functionally-dependent association of plastid gene expression. In other words, these rearrangements influence gene expression in conifers.

### Plastid RNA editing occurs in both sense and antisense transcripts

**Fig. 4:**
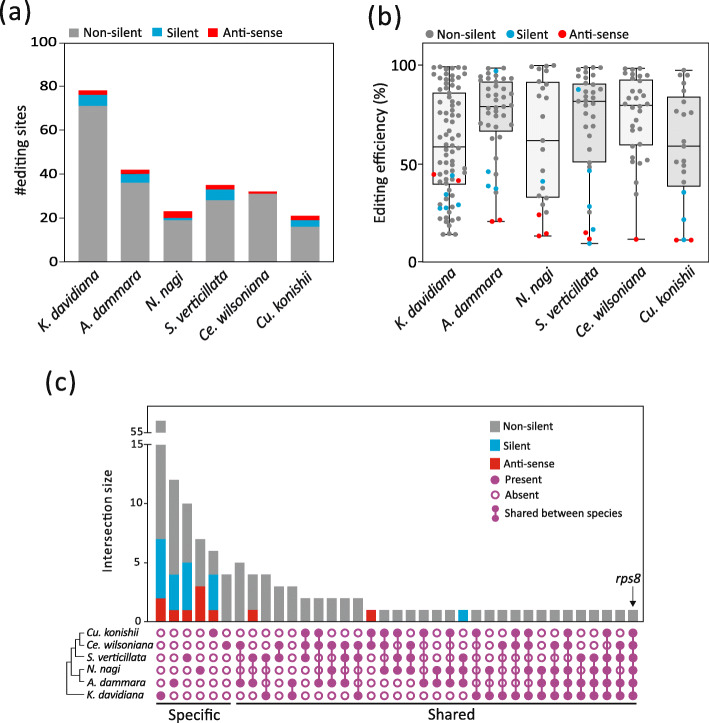
Plastid RNA-editing sites detected in the six sampled conifers. (**a**) RNA-edited sites, and (**b**) their editing efficiencies. (**c**) UpSet plot illustrating the intersections of these edited sites

We detected 78 C-to-U RNA-editing sites in *K. davidiana* plastids, 42 in *A. dammara*, 23 in *N. nagi*, 35 in *S. verticillata*, 32 in *Ce. wilsoniana*, and 21 in *Cu. konishii* (Fig. 4a; Table S1). Notably, the majority (76.2–96.9%) of these edited sites cause non-silent editing, introducing non-synonymous changes in amino acid sequences. In contrast, silent-editing sites at synonymous codon positions occur in only 0–14.3% of the sites. In addition, editing efficiency at silent-editing sites is nearly always less than 50%, with two exceptions: *ndhE* of *A. dammara* and *psbA* of *S. verticillata* (Fig. 4b; Table S1). We also discovered one to three editing sites in antisense transcripts of CDSs from each conifer species. These sites are partially edited, with efficiency less than 50% (Fig. 4b).

We further investigated the intersection among these edited sites based on their alignments. In Fig. 4c, edited sites are designated as “shared” when they appear at the same alignment position in two or more species. Those found only in a single species are designated as “specific” sites. Most silent and antisense edited sites are species-specific. Only one site—located in the *rps8* transcript—is shared by all conifers, suggesting that it originated in the common ancestor of all conifers more than 300 million years ago [20]. In addition, *K. davidiana* plastids contain more species-specific RNA-editing sites than any other species we examined, with the proportion of “specific” sites exceeding other conifers by more than twofold (Fig. S7). This finding implies that Pinaceae has evolved a distinctive set of plastid RNA-editing sites after diverging from cupressophytes.

## Discussion

We used strand-specific RNAseq data to explore plastid transcriptomic profiles across all six conifer families. Our data indicate that conifer plastomes transcribe nearly full sequences of both DNA strands, reinforcing the viewpoint that full transcription of plastomic sequences is the norm rather than an exception among seed plants [8]. We noted an excess of antisense over sense transcripts in *psbN* located at the opposite strand of the highly expressed *psbB* operon. This finding suggests that the positions of plastid genes might affect antisense RNA expression. In addition, we identified a number of C-to-U edited sites in sense and antisense transcripts. These results suggest that strand-specific RNAseq improves the detection of RNA-editing sites by not only removing antisense contamination during mapping but also allowing for the exploration of editing events in antisense transcripts. Notably, all antisense sites are edited inefficiently, implying that they are likely accidental or tissue-specific [21–23].

We also discovered numerous RNAseq reads mapping onto introns, intergenic spacers (IGSs), and the regions antisense to CDSs. Plastid non-coding RNAs were proposed to regulate gene expression [24]. In some model plants, plastid CDS and IGS transcripts have similar expression levels [7]. We did not compare transcript abundance between CDSs and IGSs because the latter’s transcriptional orientation was uncertain, making it difficult to identify the corresponding RNAseq reads in a strand-specific manner. Nonetheless, we did observe numerous transcripts antisense to CDSs. In plastids, antisense transcripts were hypothesized to bind to the 3′ end of mRNAs and stabilize them [25]. This stabilization mechanism is likely active for all CDS transcripts since their antisense counterparts are prevalent in conifer plastids.

It has long been known that transcription termination of most plastid genes is inefficient as it results in abundant and diverse read-through transcripts that must be post-transcriptionally processed [26]. In a recent study [27], the mechanism of read-through transcription, which affects the transcription of downstream genes, resulted in extreme accumulation of *accD* transcripts when transcription termination of the upstream gene, *rbcL*, was inactivated. Here, we propose that read-through transcription also helps interpret our finding that plastomic rearrangements influence gene expression. Relocating a gene involves reconfiguring its neighboring loci and thus altering the read-through transcription effect from the upstream gene. This ultimately changes the number of transcripts of the relocated gene and its downstream neighbors. Moreover, we rule out the possibility that phylogenetic effects contribute to the association between gene expression and plastomic rearrangements because the latter is not significantly correlated with the genetic distances among sampled conifers. The finding that plastomic rearrangements might influence gene expression also makes caution about determining insertion loci during transgenic experiments on highly rearranged plastomes. However, without environmental stress treatments, it is difficult to link altered gene expression from plastomic rearrangements with a biological adaption. Fortunately, inter- and intra-specific plastomic inversions have been documented in several conifer lineages [this study [18, 28–31]; providing ideal material to study the association between plastomic rearrangements and biological adaptation in the future.

## Methods

### Plant materials, DNA and RNA extraction and sequencing

The six representative conifer species (i.e., *Keteleeria davidiana* for Pinaceae, *Agathis dammara* for Araucariaceae, *Nageia nagi* for Podocarpaceae, *Sciadopitys verticillata* for Sciadopityaceae, *Cephalotaxus wilsoniana* for Taxaceae, and *Cunninghamia konishii* for Cupressaceae) were collected and identified by Dr. Chung-Shien Wu (Biodiversity Research Center, Academia Sinica). Permission was not necessary for collecting these plants. The voucher specimens were deposited at the Herbarium, Biodiversity Research Center, Academia Sinica, Taipei (HAST; Table S2).

For DNA and RNA extraction, approximately 30 cm of fresh young shoots were collected from 10 to 30 years old trees in April 2019. To reduce potential variability due to different growth conditions, shoots were grown hydroponically in a growth chamber (GC-550R, Yihder Company, New Taipei City) at 25 °C with a light intensity of 100 μmol m^− 2^ s^− 1^. After 24 h, fresh leaves on the shoots were harvested for DNA and RNA extraction using the methods described in [32, 33], respectively. The extracted DNA was sequenced at Genomics BioSci & Tech (New Taipei City, Taiwan) on an Illumina HiSeq 4000 system. We also performed strand-specific RNAseq using the same system after DNase I (Invitrogen) treatment, rRNA depletion (Illumina Ribo-Zero rRNA Removal kits, Plant Leaf version), and library construction with dUTP and random hexamers. Table S2 details the information on sampling locality, voucher numbers, GenBank accessions, and DNAseq and RNAseq read counts used in this study.

### Plastome assembly and RNA mapping analysis

Plastome assembly was initially conducted using SPAdes 3.13 [34] with the option of “careful” and a range of k-mer sizes (21, 33, 55, 77, and 99). Plastomic contigs were identified using NCBI-blast 2.2.18 [35] against in-house databases. Gaps between contigs were closed using GapCloser 1.12 [36]. This yielded complete plastomes for all sampled conifers, except *K. davidiana* because of its long Pinaceae Type I repeats [18]. We subsequently designed specific primers (Fig. S1) to amplify the corresponding regions and perform genome finishing in the latter species.

For each conifer species, 20 million paired-end RNAseq reads were randomly extracted and mapped to the corresponding plastome using TopHat 2.1.1 [37] with the parameters: library-type = fr-firststrand, read-mismatches = 15, read-gap-length = 0, and read-edit-dist = 15. Samtools 1.9 [38] was used to sort, filter, and combine the mapped reads. The resulting BAM files were imported into Geneious 11.1.5 (https://www.geneious.com) to calculate read counts and conduct downstream analyses. The RNAseq coverage, which refers to mapped read counts per base, was calculated using 100-bp non-overlapping sliding windows across plastomes, followed by transformation with the formula: Log_10_ (coverage + 1) / Log_10_ (maximum coverage + 1).

### Identification and visualization of RNA-editing sites

To identify C-to-U RNA-editing sites, the “Find Variations” option implemented in Geneious 11.1.5 was employed with the threshold: minimum coverage = 50, minimum variant frequency = 0.1, and maximum variant *P*-value = 10^− 6^. Editing efficiencies were estimated by calculating the ratio of edited to unedited bases in mapped reads. Intersection among edited sites from the six conifers were evaluated using UpSetR [39]. Plastome maps, transcriptional profiles, and RNA-editing sites were visualized using Circos 0.67 [40].

### Phylogenetic tree construction

The 83 orthologous CDSs were manually extracted from the six assembled plastomes (Table S3). Sequence alignments of these CDSs were performed using MUSCLE 3.5 [41] with the default setting. The phylogenetic tree was constructed based on the concatenation of the 83 CDSs, a GTR + G + I model, and 1000 bootstrap replicates using RAxML 8.2.10 [42].

## Conclusion

It has long been known that plastomic rearrangements occur frequently in conifers. However, gene expression dynamics in the relocated plastid gene (after rearrangements) and its downstream neighbors has not been investigated. In this pivotal study, we show that in conifers (1) both plastomic strands are fully transcribed, (2) increased plastomic arrangements reduce the strength of functionally-dependent association of plastid gene expression, (3) RNA editing occurs in both sense and antisense transcripts, and (4) the Pinaceae have evolved a distinctive set of plastid RNA-editing sites after diverging from cupressophytes. The tight association of plastomic rearrangements with gene expression leads us to propose that read-through transcription is likely the key to make this association. Additional studies and molecular biology validation are needed to better understand the biological adaptation of plastomic rearrangements in conifers.

## Supplementary Information

**Additional file 1 Table S1.** Plastid C-to-U editing sites detected in this study.

**Additional file 2 Table S2.** Conifer species, voucher numbers, DNAseq, and RNAseq data used in this study.

**Additional file 3 Table S3.** The 83 orthologous CDSs for phylogenetic tree construction in this study.

**Additional file 4 Fig. S1.** Plastomic isomers in *K. davidiana*. (a) Comparison of plastomes shows an intraspecific inversion flanked by the Pinaceae type I inverted repeat (IR). Primers used to detect specific isomers are indicated. (b) Semi-quantitative PCR demonstrates the coexistence of two isomers containing distinctive copy numbers.

**Additional file 5 Fig. S2.** Percentages of plastomic sequences covered by stranded RNAseq reads.

**Additional file 6 Fig. S3.** RNAseq coverage of CDS transcripts and their antisense counterparts in the conifer plastids. Coverage scores were transformed using Log_10_ (1 + coverage). Dashed lines denote diagonal lines. CDSs are indicated if the coverage scores of their transcripts are smaller than those of their antisense counterparts.

**Additional file 7 Fig. S4.** Extensive plastomic rearrangements in conifers. (a) Thirty-one syntenic regions (color boxes) identified in the six sampled conifer plastomes. (b) Dot-plot analyses of the six conifer plastomes.

**Additional file 8 Fig. S5.** A Pearson’s correlation test indicating that the plastomic rearrangements are not significantly correlated with the genetic distances among sampled conifers.

**Additional file 9 Fig. S6.** A Pearson’s correlation test indicating that the plastomic rearrangements are significantly and inversely correlated with the degree of the orthologous gene expression association.

**Additional file 10 Fig. S7.** Proportion of specific and shared RNA-editing sites in the six representative conifer plastids.

## Data Availability

The six plastomic data analyzed in this study are deposited into the NCBI database (https://www.ncbi.nlm.nih.gov/nucleotide/) with accession numbers: LC571739, LC571740, LC571741, LC571883, LC572146, and LC572147.

## Abbreviations

CDS: Coding sequence
IGS: Intergenic spacer
TPM: Transcripts per million
PR: Plastomic rearrangements
IR: Inverted repeat
Rho (ρ): Pearson correlation coefficient
