# Supplementary material for: Tight association of genome rearrangements with gene expression in conifer plastomes
Source: BMC Plant Biol. 2021 Jan 8;21:33. doi: 10.1186/s12870-020-02809-2 (PMC7796615; doi:10.1186/s12870-020-02809-2)
Supplement: Supplementary file 1 — Additional file 1 Table S1. Plastid C-to-U editing sites detected in this study. [file 12870_2020_2809_MOESM1_ESM.pdf]

Table S1. Plastid C-to-U editing sites detected in this study

Species	Editing type	CDS	CDS position	Codon position	Editing efficiency
Agathis dammara	Silent	*atpB*	1,413	3	39.10%
Agathis dammara	Non-silent	*atpF*	23	2	83.80%
Agathis dammara	Non-silent	*atpI*	617	2	95.80%
Agathis dammara	Non-silent	*atpI*	431	2	98.50%
Agathis dammara	Non-silent	*ccsA*	404	2	93.10%
Agathis dammara	Antisense	*matK*	1,534	-	21.20%
Agathis dammara	Non-silent	*matK*	895	1	75.80%
Agathis dammara	Non-silent	*ndhA*	47	2	78.30%
Agathis dammara	Non-silent	*ndhA*	482	2	85.00%
Agathis dammara	Non-silent	*ndhB*	776	2	92.30%
Agathis dammara	Non-silent	*ndhB*	1,385	2	94.20%
Agathis dammara	Non-silent	*ndhD*	272	2	68.80%
Agathis dammara	Non-silent	*ndhE*	142	1	93.30%
Agathis dammara	Silent	*ndhE*	180	3	97.00%
Agathis dammara	Non-silent	*ndhE*	197	2	89.40%
Agathis dammara	Non-silent	*ndhF*	1,802	2	85.50%
Agathis dammara	Non-silent	*ndhF*	1,841	2	88.00%
Agathis dammara	Non-silent	*ndhF*	2,039	2	93.30%
Agathis dammara	Antisense	*ndhF*	2,026	-	21.90%
Agathis dammara	Non-silent	*ndhH*	1,094	2	74.60%
Agathis dammara	Non-silent	*psbH*	173	2	98.30%
Agathis dammara	Non-silent	*psbM*	47	2	94.70%
Agathis dammara	Non-silent	*psbN*	37	1	87.20%
Agathis dammara	Non-silent	*rpl20*	269	2	79.10%
Agathis dammara	Non-silent	*rpoB*	2,912	2	63.70%
Agathis dammara	Non-silent	*rpoB*	2,495	2	45.00%
Agathis dammara	Non-silent	*rpoB*	473	2	35.80%
Agathis dammara	Non-silent	*rpoC1*	1,061	2	82.40%
Agathis dammara	Non-silent	*rpoC1*	470	2	74.30%
Agathis dammara	Non-silent	*rpoC1*	419	2	63.00%
Agathis dammara	Silent	*rpoC1*	168	3	46.30%
Agathis dammara	Non-silent	*rpoC1*	164	2	84.60%
Agathis dammara	Non-silent	*rpoC2*	2,909	2	78.60%
Agathis dammara	Non-silent	*rpoC2*	155	2	69.90%
Agathis dammara	Non-silent	*rps14*	149	2	91.60%
Agathis dammara	Silent	*rps8*	240	3	37.80%

Agathis dammara	Non-silent	*rps8*	203	2	70.60%
Agathis dammara	Non-silent	*rps8*	161	2	79.70%
Agathis dammara	Non-silent	*rps8*	155	2	76.70%
Agathis dammara	Non-silent	*rps8*	145	1	69.70%
Agathis dammara	Non-silent	*ycf1*	6,290	2	53.00%
Agathis dammara	Non-silent	*ycf2*	4,838	2	88.00%
Cephalotaxus wilsoniana	Non-silent	*atpA*	773	2	95.90%
Cephalotaxus wilsoniana	Non-silent	*atpI*	614	2	93.30%
Cephalotaxus wilsoniana	Antisense	*chlB*	18	-	12.20%
Cephalotaxus wilsoniana	Non-silent	*chlN*	850	1	94.40%
Cephalotaxus wilsoniana	Non-silent	*matK*	892	1	69.30%
Cephalotaxus wilsoniana	Non-silent	*matK*	1,358	2	53.40%
Cephalotaxus wilsoniana	Non-silent	*ndhB*	28	1	60.10%
Cephalotaxus wilsoniana	Non-silent	*ndhB*	776	2	83.30%
Cephalotaxus wilsoniana	Non-silent	*ndhD*	1,193	2	94.80%
Cephalotaxus wilsoniana	Non-silent	*ndhE*	293	2	95.40%
Cephalotaxus wilsoniana	Non-silent	*ndhF*	704	2	84.60%
Cephalotaxus wilsoniana	Non-silent	*ndhF*	1,808	2	86.80%
Cephalotaxus wilsoniana	Non-silent	*ndhF*	1,847	2	86.80%
Cephalotaxus wilsoniana	Non-silent	*petG*	44	2	50.50%
Cephalotaxus wilsoniana	Non-silent	*petL*	74	2	80.40%
Cephalotaxus wilsoniana	Non-silent	*psaA*	725	2	97.80%
Cephalotaxus wilsoniana	Non-silent	*psaC*	146	2	98.20%
Cephalotaxus wilsoniana	Non-silent	*psbH*	173	2	98.30%
Cephalotaxus wilsoniana	Non-silent	*psbM*	47	2	72.20%
Cephalotaxus wilsoniana	Non-silent	*psbN*	37	1	79.20%
Cephalotaxus wilsoniana	Non-silent	*rpoB*	920	2	67.10%
Cephalotaxus wilsoniana	Non-silent	*rpoB*	884	2	67.80%
Cephalotaxus wilsoniana	Non-silent	*rpoB*	839	2	52.10%
Cephalotaxus wilsoniana	Non-silent	*rpoB*	473	2	35.00%
Cephalotaxus wilsoniana	Non-silent	*rpoC1*	479	2	83.30%
Cephalotaxus wilsoniana	Non-silent	*rpoC2*	2,867	2	80.40%
Cephalotaxus wilsoniana	Non-silent	*rpoC2*	2,615	2	78.60%
Cephalotaxus wilsoniana	Non-silent	*rpoC2*	176	2	40.80%
Cephalotaxus wilsoniana	Non-silent	*rps8*	172	1	51.30%
Cephalotaxus wilsoniana	Non-silent	*rps8*	188	2	59.10%
Cephalotaxus wilsoniana	Non-silent	*rps8*	230	2	92.00%
Cephalotaxus wilsoniana	Non-silent	*ycf3*	191	2	76.80%

Cunninghamia konishii	Non-silent	*atpA*	773	2	94.90%
Cunninghamia konishii	Antisense	*atpA*	184	-	11.80%
Cunninghamia konishii	Antisense	*chlB*	18	-	11.80%
Cunninghamia konishii	Non-silent	*ndhB*	776	2	90.90%
Cunninghamia konishii	Non-silent	*ndhB*	1,127	2	81.50%
Cunninghamia konishii	Non-silent	*ndhE*	293	2	97.40%
Cunninghamia konishii	Non-silent	*ndhF*	25	1	95.00%
Cunninghamia konishii	Non-silent	*ndhH*	1,094	2	59.10%
Cunninghamia konishii	Silent	*petN*	12	3	22.20%
Cunninghamia konishii	Non-silent	*psbZ*	149	2	46.20%
Cunninghamia konishii	Silent	*rpoB*	390	3	12.00%
Cunninghamia konishii	Non-silent	*rpoB*	917	2	51.70%
Cunninghamia konishii	Non-silent	*rpoB*	962	2	76.00%
Cunninghamia konishii	Non-silent	*rpoB*	1,486	1	51.20%
Cunninghamia konishii	Non-silent	*rpoC1*	584	2	41.00%
Cunninghamia konishii	Non-silent	*rpoC2*	2,351	2	75.10%
Cunninghamia konishii	Non-silent	*rps14*	149	2	76.70%
Cunninghamia konishii	Non-silent	*rps8*	188	2	49.40%
Cunninghamia konishii	Non-silent	*rps8*	230	2	87.00%
Cunninghamia konishii	Silent	*ycf1*	6,850	1	35.90%
Cunninghamia konishii	Non-silent	*ycf3*	191	2	61.50%
Keteleeria davidiana	Non-silent	*accD*	935	2	81.60%
Keteleeria davidiana	Non-silent	*atpA*	554	2	94.70%
Keteleeria davidiana	Non-silent	*atpA*	773	2	88.60%
Keteleeria davidiana	Non-silent	*atpA*	1,004	2	95.60%
Keteleeria davidiana	Non-silent	*atpB*	1,049	2	76.00%
Keteleeria davidiana	Non-silent	*atpB*	1,217	2	97.90%
Keteleeria davidiana	Non-silent	*atpF*	65	2	71.40%
Keteleeria davidiana	Non-silent	*atpF*	101	2	59.40%
Keteleeria davidiana	Non-silent	*atpF*	370	1	56.30%
Keteleeria davidiana	Non-silent	*atpI*	152	2	92.70%
Keteleeria davidiana	Non-silent	*atpI*	428	2	77.20%
Keteleeria davidiana	Non-silent	*atpI*	614	2	90.70%
Keteleeria davidiana	Non-silent	*ccsA*	716	2	74.70%
Keteleeria davidiana	Non-silent	*ccsA*	704	2	81.30%
Keteleeria davidiana	Non-silent	*ccsA*	407	2	79.60%
Keteleeria davidiana	Non-silent	*cemA*	701	2	80.70%
Keteleeria davidiana	Non-silent	*cemA*	116	2	14.70%

Keteleeria davidiana	Non-silent	*chlB*	1,280	2	22.60%
Keteleeria davidiana	Non-silent	*chlB*	646	1	46.00%
Keteleeria davidiana	Non-silent	*chlB*	13	1	29.80%
Keteleeria davidiana	Non-silent	*chlN*	856	1	15.10%
Keteleeria davidiana	Silent	*chlN*	1,005	3	28.10%
Keteleeria davidiana	Non-silent	*clpP*	194	2	87.70%
Keteleeria davidiana	Non-silent	*matK*	1,466	2	19.10%
Keteleeria davidiana	Non-silent	*matK*	892	1	34.30%
Keteleeria davidiana	Non-silent	*matK*	527	2	72.10%
Keteleeria davidiana	Non-silent	*petB*	155	2	93.40%
Keteleeria davidiana	Non-silent	*petB*	599	2	59.90%
Keteleeria davidiana	Non-silent	*petB*	634	1	64.90%
Keteleeria davidiana	Non-silent	*petG*	80	2	74.60%
Keteleeria davidiana	Non-silent	*petG*	17	2	89.80%
Keteleeria davidiana	Non-silent	*petL*	74	2	92.40%
Keteleeria davidiana	Non-silent	*psaI*	79	1	83.90%
Keteleeria davidiana	Non-silent	*psbB*	1,391	2	98.60%
Keteleeria davidiana	Non-silent	*psbC*	1,211	2	99.10%
Keteleeria davidiana	Non-silent	*psbC*	524	2	88.40%
Keteleeria davidiana	Non-silent	*psbD*	935	2	98.40%
Keteleeria davidiana	Non-silent	*psbD*	839	2	98.80%
Keteleeria davidiana	Non-silent	*psbD*	398	2	98.80%
Keteleeria davidiana	Non-silent	*psbE*	214	1	85.80%
Keteleeria davidiana	Silent	*psbK*	93	3	34.80%
Keteleeria davidiana	Non-silent	*psbK*	92	2	95.50%
Keteleeria davidiana	Non-silent	*psbZ*	149	2	95.40%
Keteleeria davidiana	Non-silent	*psbZ*	20	2	47.90%
Keteleeria davidiana	Non-silent	*rpl2*	515	2	42.40%
Keteleeria davidiana	Non-silent	*rpl2*	203	2	75.80%
Keteleeria davidiana	Non-silent	*rpl23*	61	1	42.40%
Keteleeria davidiana	Non-silent	*rpl32*	58	1	93.70%
Keteleeria davidiana	Silent	*rpoA*	100	1	44.40%
Keteleeria davidiana	Non-silent	*rpoB*	397	1	21.80%
Keteleeria davidiana	Non-silent	*rpoB*	545	2	50.90%
Keteleeria davidiana	Non-silent	*rpoB*	815	2	28.50%
Keteleeria davidiana	Non-silent	*rpoB*	1,841	2	62.90%
Keteleeria davidiana	Non-silent	*rpoB*	2,444	2	46.80%
Keteleeria davidiana	Non-silent	*rpoB*	2,722	1	23.10%

Keteleeria davidiana	Non-silent	*rpoB*	2,861	2	51.60%
Keteleeria davidiana	Non-silent	*rpoC1*	164	2	32.60%
Keteleeria davidiana	Non-silent	*rpoC1*	479	2	66.90%
Keteleeria davidiana	Non-silent	*rpoC1*	875	2	22.60%
Keteleeria davidiana	Non-silent	*rpoC1*	1,487	2	41.20%
Keteleeria davidiana	Non-silent	*rpoC2*	937	1	21.10%
Keteleeria davidiana	Non-silent	*rpoC2*	2,729	2	45.80%
Keteleeria davidiana	Non-silent	*rpoC2*	2,981	2	63.60%
Keteleeria davidiana	Non-silent	*rpoC2*	3,254	2	64.60%
Keteleeria davidiana	Antisense	*rpoC2*	1,968	-	41.80%
Keteleeria davidiana	Non-silent	*rps14*	146	2	49.70%
Keteleeria davidiana	Silent	*rps2*	222	3	27.80%
Keteleeria davidiana	Non-silent	*rps2*	344	2	54.70%
Keteleeria davidiana	Non-silent	*rps2*	662	2	57.40%
Keteleeria davidiana	Non-silent	*rps4*	272	2	50.80%
Keteleeria davidiana	Non-silent	*rps8*	254	2	45.00%
Keteleeria davidiana	Non-silent	*rps8*	188	2	40.40%
Keteleeria davidiana	Non-silent	*rps8*	182	2	37.00%
Keteleeria davidiana	Silent	*ycf1*	4,131	3	29.60%
Keteleeria davidiana	Antisense	*ycf2*	3,107	-	44.90%
Keteleeria davidiana	Non-silent	*ycf2*	5,003	2	25.90%
Keteleeria davidiana	Non-silent	*ycf2*	4,723	1	58.20%
Keteleeria davidiana	Non-silent	*ycf2*	569	2	14.70%
Nageia nagi	Non-silent	*atpA*	773	2	99.80%
Nageia nagi	Antisense	*atpI*	403	-	15.10%
Nageia nagi	Antisense	*atpI*	31	-	24.60%
Nageia nagi	Non-silent	*atpI*	617	2	99.50%
Nageia nagi	Non-silent	*ndhD*	1,193	2	98.10%
Nageia nagi	Non-silent	*ndhF*	25	1	57.50%
Nageia nagi	Non-silent	*ndhH*	1,088	2	91.70%
Nageia nagi	Antisense	*ndhI*	48	-	13.90%
Nageia nagi	Non-silent	*rpoA*	299	2	90.30%
Nageia nagi	Non-silent	*rpoB*	2,894	2	46.80%
Nageia nagi	Non-silent	*rpoB*	470	2	38.00%
Nageia nagi	Non-silent	*rpoC1*	1,085	2	34.10%
Nageia nagi	Non-silent	*rpoC2*	2,822	2	88.30%
Nageia nagi	Non-silent	*rps14*	149	2	99.20%
Nageia nagi	Non-silent	*rps8*	172	1	74.10%

Nageia nagi	Non-silent	*rps8*	182	2	84.40%
Nageia nagi	Non-silent	*rps8*	188	2	83.40%
Nageia nagi	Silent	*rps8*	267	3	41.40%
Nageia nagi	Non-silent	*rps8*	274	1	33.00%
Nageia nagi	Non-silent	*rps8*	284	2	25.80%
Nageia nagi	Non-silent	*ycf1*	455	2	29.70%
Nageia nagi	Non-silent	*ycf2*	4,733	2	97.10%
Nageia nagi	Non-silent	*ycf3*	191	2	62.00%
Sciadopitys verticillata	Non-silent	*ccsA*	407	2	81.90%
Sciadopitys verticillata	Non-silent	*chlB*	13	1	48.60%
Sciadopitys verticillata	Non-silent	*chlN*	850	1	83.50%
Sciadopitys verticillata	Non-silent	*matK*	892	1	79.80%
Sciadopitys verticillata	Non-silent	*ndhA*	23	2	80.60%
Sciadopitys verticillata	Non-silent	*ndhA*	338	2	25.90%
Sciadopitys verticillata	Non-silent	*ndhA*	482	2	81.80%
Sciadopitys verticillata	Non-silent	*ndhD*	272	2	87.80%
Sciadopitys verticillata	Non-silent	*ndhD*	1,193	2	93.60%
Sciadopitys verticillata	Non-silent	*ndhE*	293	2	90.20%
Sciadopitys verticillata	Antisense	*ndhF*	2,026	-	15.60%
Sciadopitys verticillata	Non-silent	*ndhF*	2,039	2	90.90%
Sciadopitys verticillata	Non-silent	*ndhF*	1,802	2	86.20%
Sciadopitys verticillata	Non-silent	*ndhF*	25	1	64.30%
Sciadopitys verticillata	Silent	*ndhH*	1,158	3	46.80%
Sciadopitys verticillata	Non-silent	*petD*	134	2	98.80%
Sciadopitys verticillata	Non-silent	*psaA*	725	2	97.70%
Sciadopitys verticillata	Non-silent	*psaC*	146	2	98.70%
Sciadopitys verticillata	Silent	*psbA*	696	3	87.70%
Sciadopitys verticillata	Non-silent	*psbC*	523	1	75.60%
Sciadopitys verticillata	Non-silent	*psbC*	524	2	94.90%
Sciadopitys verticillata	Non-silent	*psbE*	214	1	96.40%
Sciadopitys verticillata	Non-silent	*psbK*	92	2	98.70%
Sciadopitys verticillata	Silent	*rbcL*	93	3	10.10%
Sciadopitys verticillata	Antisense	*rpl33*	135	-	12.40%
Sciadopitys verticillata	Non-silent	*rpoB*	887	2	74.00%
Sciadopitys verticillata	Non-silent	*rpoB*	923	2	69.20%
Sciadopitys verticillata	Non-silent	*rpoB*	3,044	2	68.90%
Sciadopitys verticillata	Silent	*rpoC2*	2,944	1	17.20%
Sciadopitys verticillata	Non-silent	*rpoC2*	2,624	2	86.60%

Sciadopitys verticillata	Non-silent	*rpoC2*	152	2	84.10%
Sciadopitys verticillata	Non-silent	*rps14*	149	2	94.40%
Sciadopitys verticillata	Non-silent	*rps8*	155	2	50.80%
Sciadopitys verticillata	Non-silent	*rps8*	161	2	57.50%
Sciadopitys verticillata	Silent	*rps8*	237	3	28.80%
