# Supplementary material for: Tight association of genome rearrangements with gene expression in conifer plastomes
Source: BMC Plant Biol. 2021 Jan 8;21:33. doi: 10.1186/s12870-020-02809-2 (PMC7796615; doi:10.1186/s12870-020-02809-2)
Supplement: Supplementary file 2 — Additional file 2 Table S2. Conifer species, voucher numbers, DNAseq, and RNAseq data used in this study. [file 12870_2020_2809_MOESM2_ESM.pdf]

Table S2. Conifer species, sampling locality, voucher numbers, DNAseq, and RNAseq data used in this study

Family	Species	Sampling locality	Voucher number	GenBank accession	DNAseq*	RNAseq*
Pinaceae	*Keteleeria davidiana*	Taipei Botanical Garden, Taiwan	Chaw 1470	LC571883	16.8 Gb	12.6 Gb
Araucariaceae	*Agathis dammara*	Taipei Botanical Garden, Taiwan	Chaw 1490	LC571739	8.1 Gb	12.8 Gb
Podocarpaceae	*Nageia nagi*	Academia Sinica, Taiwan	Chaw 1491	LC572146	11.1 Gb	12.7 Gb
Sciadopityaceae	*Sciadopitys verticillata*	Taipei Floriculture Experiment Center, Taiwan	Chaw 1497	LC572147	14.6 Gb	12.8 Gb
Taxaceae	*Cephalotaxus wilsoniana*	Taipei Botanical Garden, Taiwan	Chaw 1492	LC571740	15.6 Gb	12.8 Gb
Cupressaceae	*Cunninghamia konishii*	Academia Sinica, Taiwan	Chaw 1438	LC571741	16.5 Gb	12.7 Gb

*150-bp paired-end reads after quality trimming
