# Supplementary material for: Tight association of genome rearrangements with gene expression in conifer plastomes
Source: BMC Plant Biol. 2021 Jan 8;21:33. doi: 10.1186/s12870-020-02809-2 (PMC7796615; doi:10.1186/s12870-020-02809-2)
Supplement: Supplementary file 3 — Additional file 3 Table S3. The 83 orthologous CDSs for phylogenetic tree construction in this study. [file 12870_2020_2809_MOESM3_ESM.pdf]

Table S3. Plastid CDSs used in phylogenetic tree construction

accD, *atpA*, *atpB*, *atpE*, *atpF*, *atpH*, *atpI*, *ccsA*, *cemA*, *chlB*, *chlL*, *chlN*, *clpP*, *infA*, *matK*, *ndhA*, *ndhB*, *ndhC*, *ndhD*, *ndhE*, *ndhF*, *ndhG*, *ndhH*, *ndhI*, *ndhJ*, *ndhK*, *petA*, *petB*, *petD*, *petG*, *petL*, *petN*, *psaA*, *psaB*, *psaC*, *psaI*, *psaJ*, *psaM*, *psbA*, *psbB*, *psbC*, *psbD*, *psbE*, *psbF*, *psbH*, *psbI*, *psbJ*, *psbK*, *psbL*, *psbM*, *psbN*, *psbT*, *psbZ*, *rbcL*, *rpl2*, *rpl14*, *rpl16*, *rpl20*, *rpl22*, *rpl23*, *rpl32*, *rpl33*, *rpl36*, *rpoA*, *rpoB*, *rpoC1*, *rpoC2*, *rps2*, *rps3*, *rps4*, *rps7*, *rps8*, *rps11*, *rps12*, *rps14*, *rps15*, *rps16*, *rps18*, *rps19*, *ycf1*, *ycf2*, *ycf3*, *ycf4*
