# Supplementary figures and images for: Tight association of genome rearrangements with gene expression in conifer plastomes
Source: BMC Plant Biol. 2021 Jan 8;21:33. doi: 10.1186/s12870-020-02809-2 (PMC7796615; doi:10.1186/s12870-020-02809-2)

Figure S1

(a)

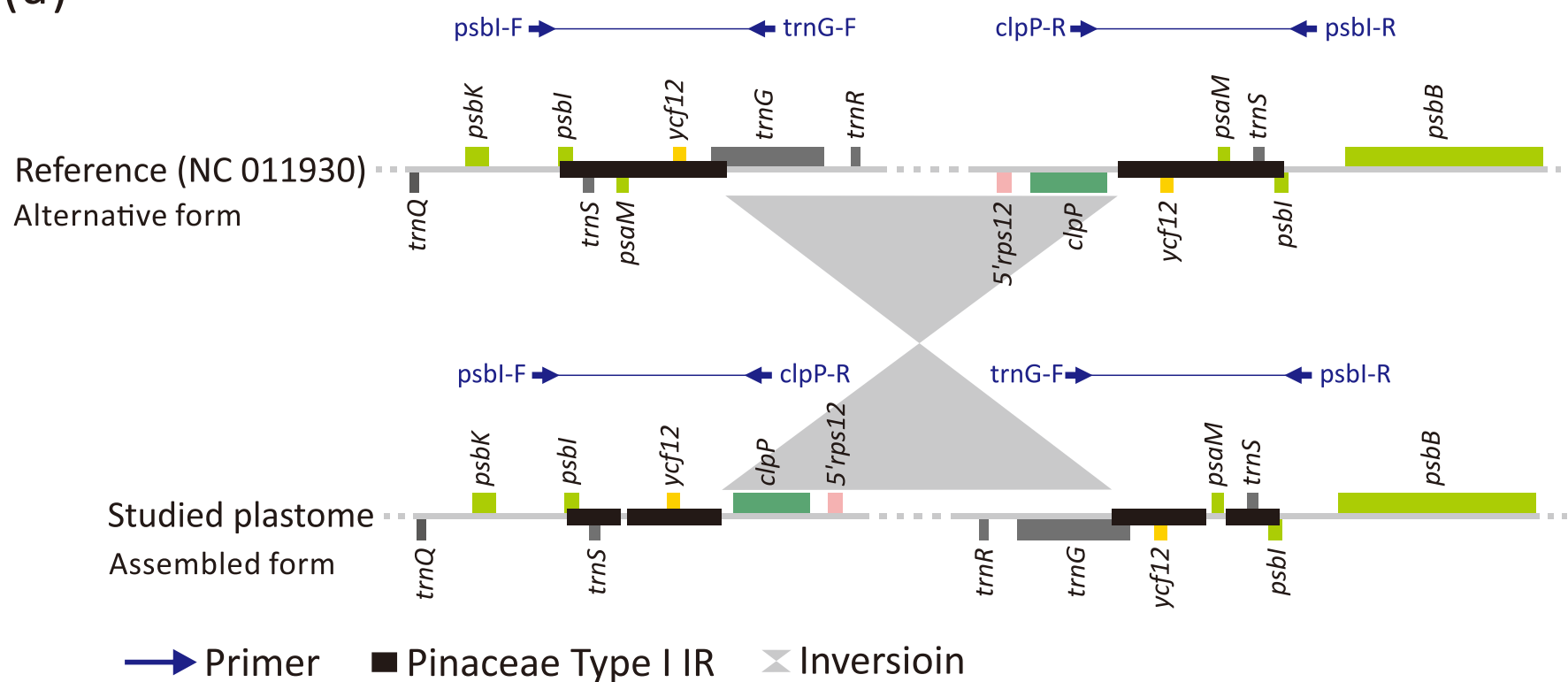

(b)

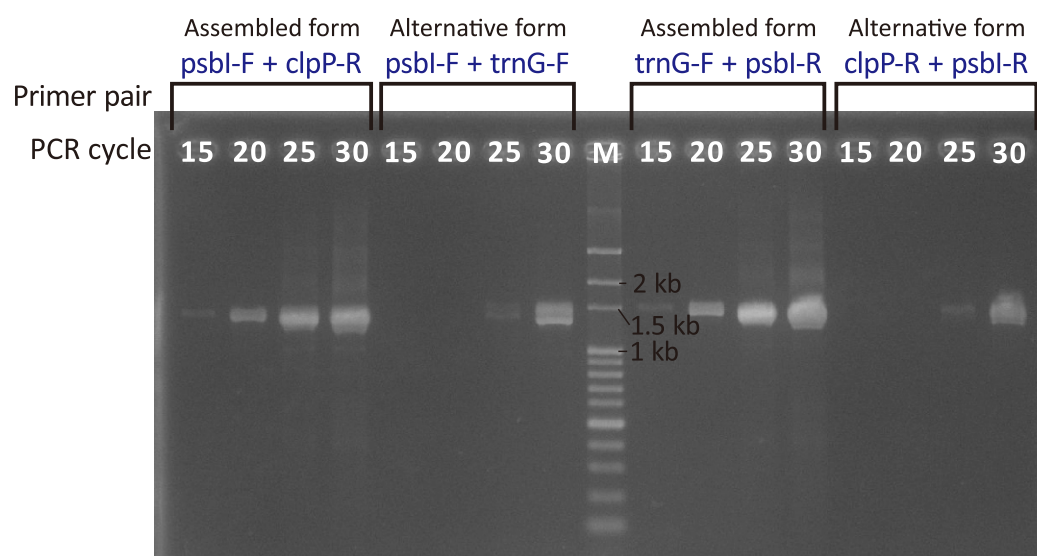

Supplement: Supplementary file 4 — Additional file 4 Fig. S1. Plastomic isomers in K. davidiana. (a) Comparison of plastomes shows an intraspecific inversion flanked by the Pinaceae type I inverted repeat (IR). Primers used to detect specific isomers are indicated. (b) Semi-quantitative PCR demonstrates the coexistence of two isomers containing distinctive copy numbers. [file 12870_2020_2809_MOESM4_ESM.pdf]

Figure S2

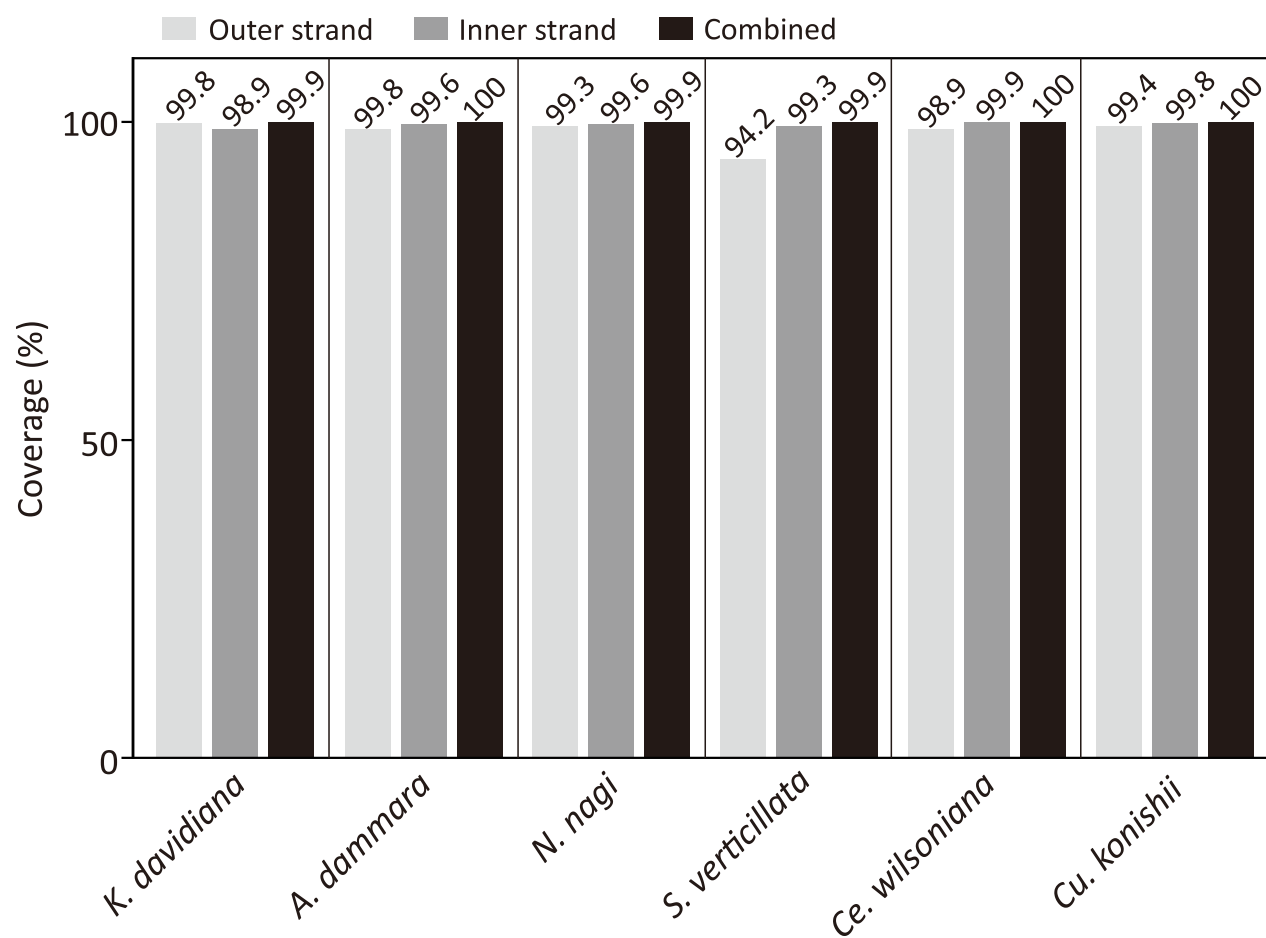

Supplement: Supplementary file 5 — Additional file 5 Fig. S2. Percentages of plastomic sequences covered by stranded RNAseq reads. [file 12870_2020_2809_MOESM5_ESM.pdf]

Figure S3

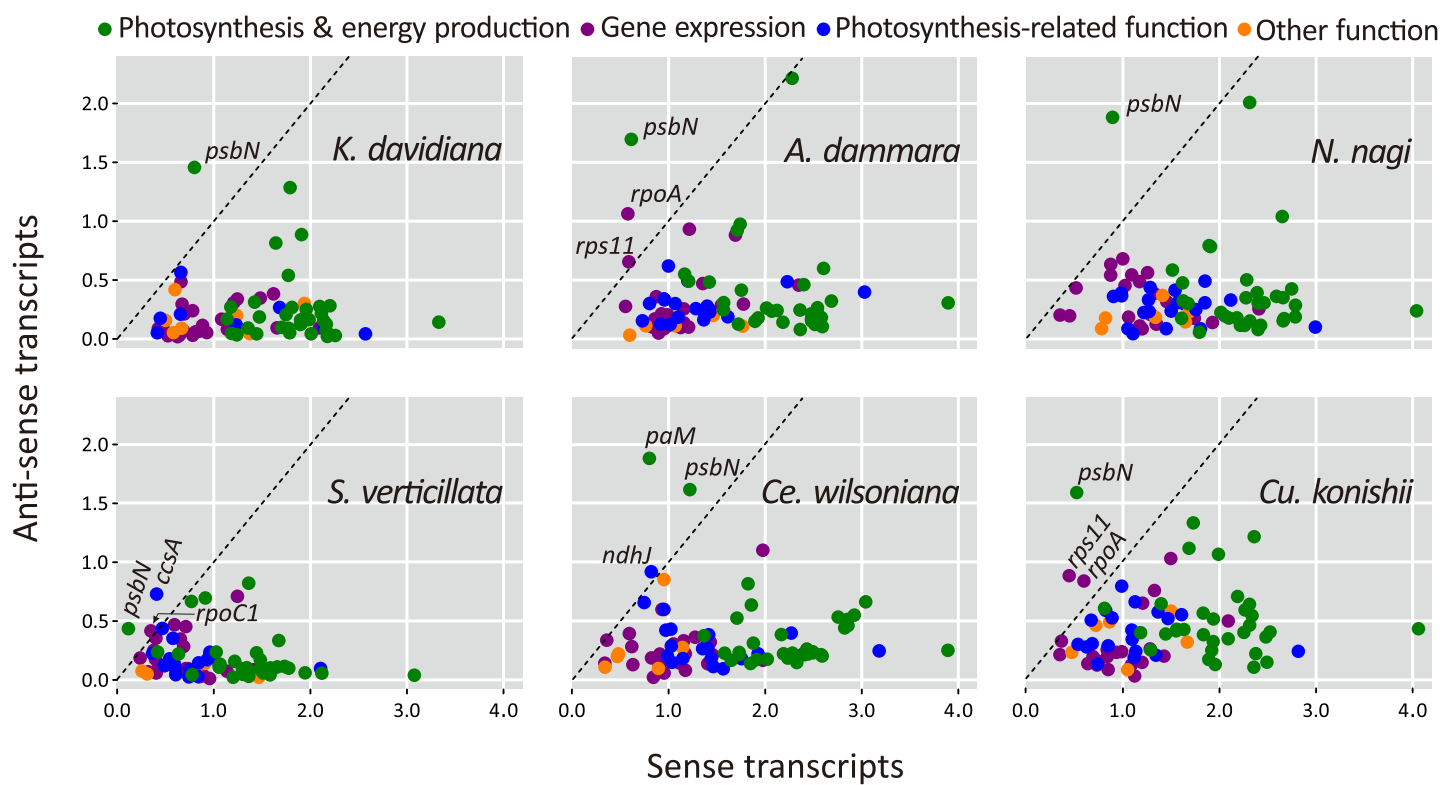

Supplement: Supplementary file 6 — Additional file 6 Fig. S3. RNAseq coverage of CDS transcripts and their antisense counterparts in the conifer plastids. Coverage scores were transformed using Log10 (1 + coverage). Dashed lines denote diagonal lines. CDSs are indicated if the coverage scores of their transcripts are smaller than those of their antisense counterparts. [file 12870_2020_2809_MOESM6_ESM.pdf]

Figure S4

(a)

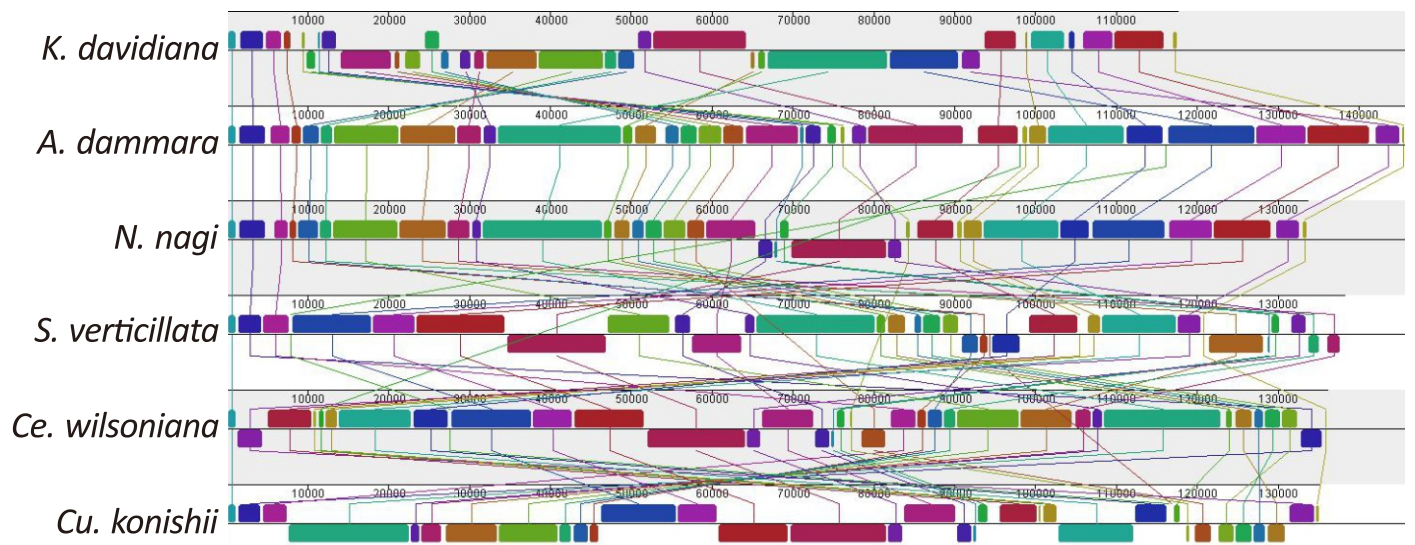

(b)

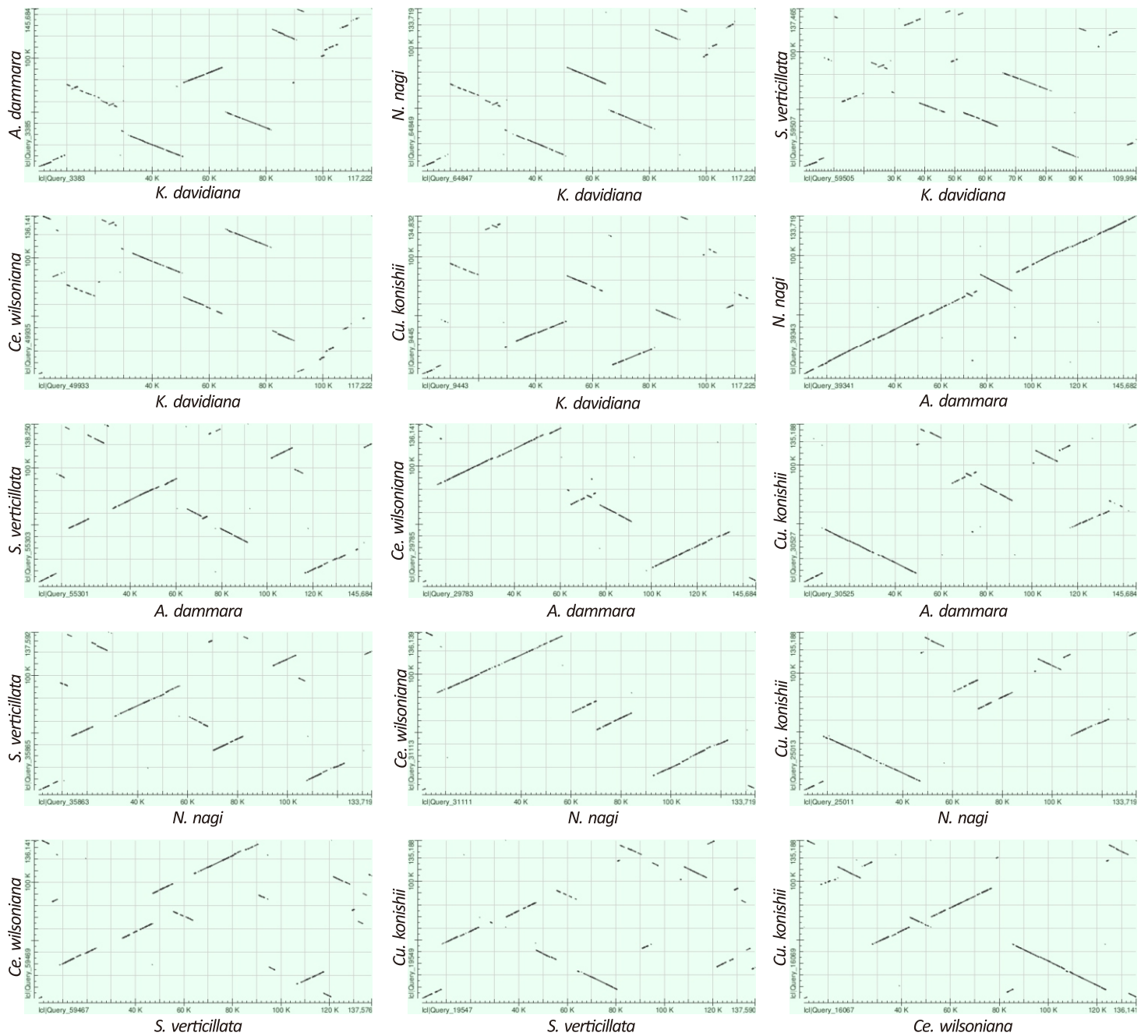

Supplement: Supplementary file 7 — Additional file 7 Fig. S4. Extensive plastomic rearrangements in conifers. (a) Thirty-one syntenic regions (color boxes) identified in the six sampled conifer plastomes. (b) Dot-plot analyses of the six conifer plastomes. [file 12870_2020_2809_MOESM7_ESM.pdf]

Figure S5

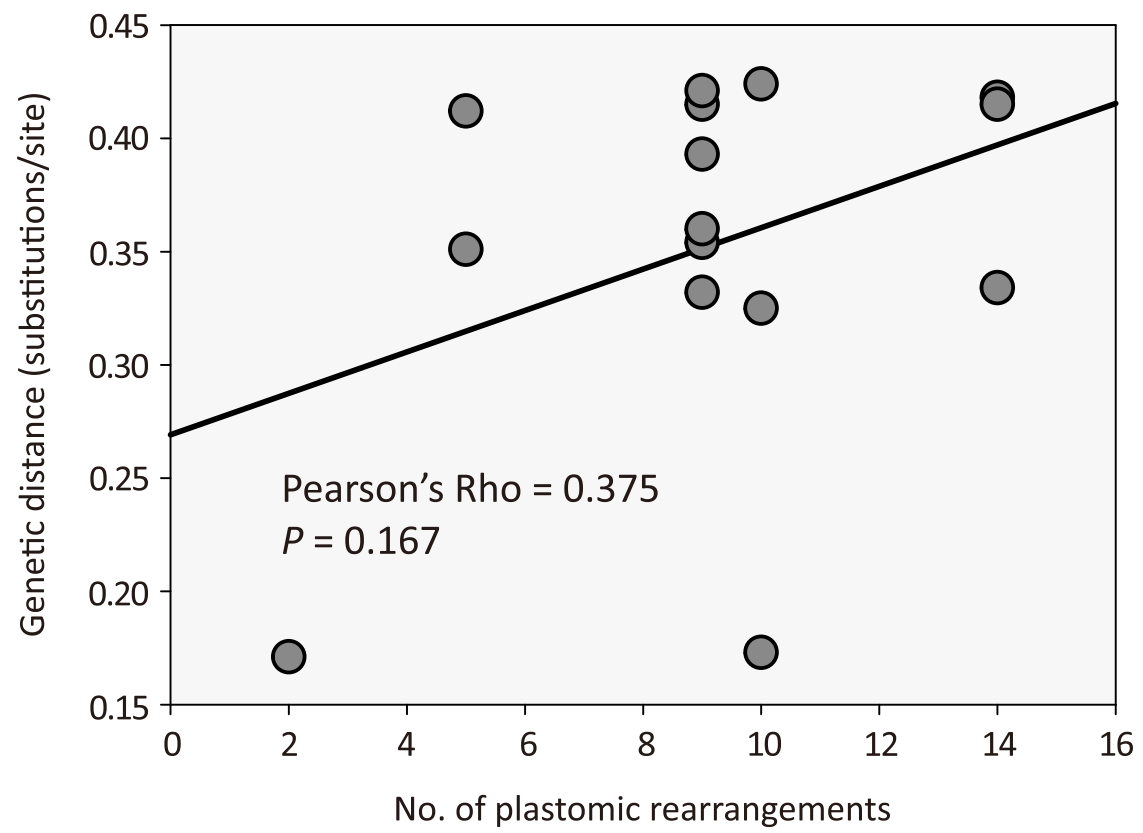

Supplement: Supplementary file 8 — Additional file 8 Fig. S5. A Pearson’s correlation test indicating that the plastomic rearrangements are not significantly correlated with the genetic distances among sampled conifers. [file 12870_2020_2809_MOESM8_ESM.pdf]

Figure S6

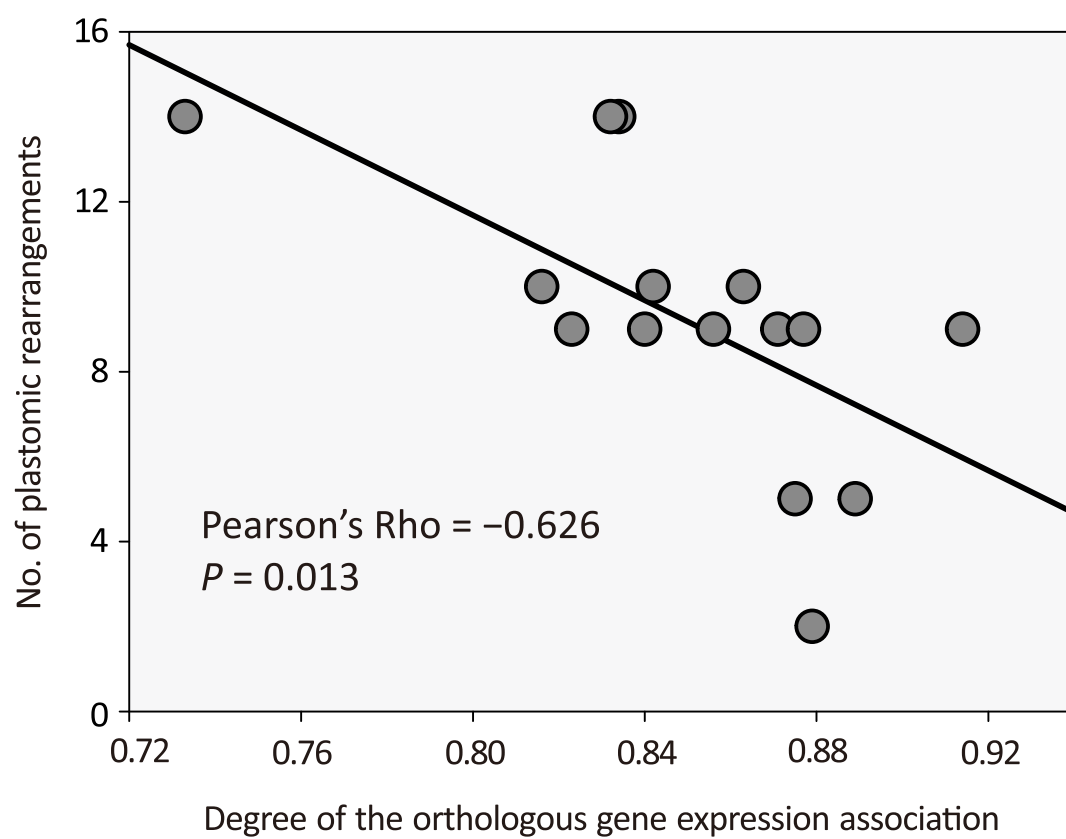

Supplement: Supplementary file 9 — Additional file 9 Fig. S6. A Pearson’s correlation test indicating that the plastomic rearrangements are significantly and inversely correlated with the degree of the orthologous gene expression association. [file 12870_2020_2809_MOESM9_ESM.pdf]

Figure S7

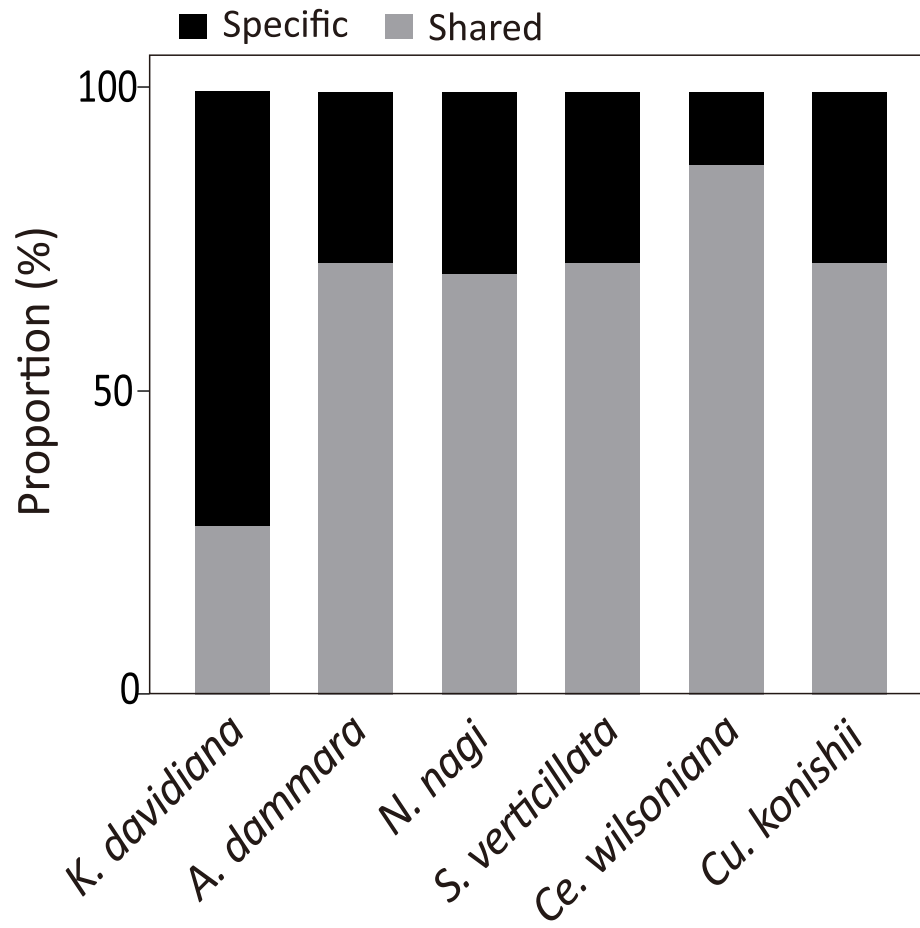

Supplement: Supplementary file 10 — Additional file 10 Fig. S7. Proportion of specific and shared RNA-editing sites in the six representative conifer plastids. [file 12870_2020_2809_MOESM10_ESM.pdf]
